# Revenue for Conducting the Work of State Boards of Dental Examiners

**Published:** 1901-07

**Authors:** George L. Parmele

**Affiliations:** Hartford, Conn.


(c) BY TAXATION OF THE PROFESSION.

### REVENUE FOR CONDUCTING THE WORK OF STATE BOARDS OF DENTAL EXAMINERS.[1]

BY DR. GEORGE L. PARMELE, HARTFORD, CONN.

In this "Symposium on State Boards of Dental Examiners in their Relations to the Profession and the Colleges," I am asked to deal simply with this subdivision: "How shall the Revenue for conducting the Work of the Boards of Examiners be obtained?"

(*a*) By taxation of the people.

---

[1] Abstract of paper read before the American Medical Association, Section on Stomatology, St. Paul, Minn., June 4, 5, 6, and 7, 1901.

(*b*) By fees from the candidates.

(*c*) By taxation of the profession.

Naturally our first inquiry would be as to the causes bringing about dental legislation, and the purpose of such laws.

The majority of the dental world labor under the idea that these dental laws were passed to protect the dentist from the dentist; that is, to bar out the dental parlor man and admit to practice only those who will live up to the code of ethics; but notwithstanding the fact that more or less selfishness on the part of the dental profession prompted their advocacy of such enactments, the laws are for the safety and welfare of the people.

This being the case, you say, let the people pay.

No doubt there are many good reasons why the people should pay; for example, were the people taxed, then many worthy but financially embarrassed young candidates, who have expended their last hard-earned dollar at college, would not be required to wait until they could earn enough to satisfy the demands of the State boards, although I think all candidates would be better off if they would serve as assistants with some successful practitioner. There they would gain experience, not only in the manipulation necessary to the care of the dental organs, but learn, what is vastly of more importance to them at the outset, how to deal with people and to meet the many petty but important details of daily routine in a successful professional office.

Let us leave class "*b*," "fees from the candidate," while we quickly dispose of class "*c*," "taxation of the profession."

In our professional ranks there are many generous, whole-souled, self-sacrificing men, but when we talk of securing the revenue by taxing the profession, it means the whole profession, and, judging from my own experience with the average dentist, it would be a greater task to get the dental profession to allow itself to be taxed, so that other dentists might enter into competition with them, than it would be to get the people, the free and enlightened voter, to submit to taxation in order that another voter might carry on his business.

This brings us to the only other source of revenue, "*b*," "the candidate."

When I was requested to prepare this paper there came upon me a sudden elation, for it seemed that for once I knew where to go for the desired information.

In a chat with a lawyer along the lines of dental legislation, he gave me the impression that the State should pay the costs of examination, but alas! when I hastened to him, note-book in hand, I found that I had misunderstood his remarks. Our talk had been of a certain retroactive clause in a proposed dental bill, and that which he had intended to say was, that if this bill should pass it would be a hardship to compel certain ones to pay a second fee, and, in justice, the State in this case should be responsible.

Continuing the conversation after I had stated the question I was to deal with, the attorney assured me that so far as he was informed of the various license granting powers, there could be but one answer to the question.

Later investigation has convinced me of the soundness of his opinion.

In the work of various other boards,—for example, health, forestry, fisheries,—the expenses justly come from the public funds, as the benefit is not for individuals, but for the people; but in the case of a license to carry on some trade or profession, whereby an individual is allowed certain privileges, the individual should pay the costs, notwithstanding the fact that the laws governing such cases are enacted for public protection.

Let us glance for a moment along the various walks in life where licenses are required before one can enter their ranks, and see, by actual reference to a few State laws, from what source the revenue is obtained.

Georgia, State Board of Embalmers, license five dollars, from the candidate.

Ohio, physicians, dentists, and druggists, fee twenty-five dollars, from the candidate.

South Carolina, Homœopathic Board, fee five dollars, from the candidate.

New Hampshire, plumbers, fee one dollar for examination, fifty cents every year, from the candidate.

Virginia, medicine and surgery, fee ten dollars, from the candidate.

Nebraska, barbers, examination one dollar, and annually one dollar, from the candidate.

Michigan, barbers, examination five dollars, and annually fifty cents, from the candidate. Barbers in business at passage of law, one dollar, and annually fifty cents.

Tennessee, osteopathy, fee from candidate for recording diploma from one named college, one dollar; all not so recorded, fine one hundred dollars.

This list could be continued indefinitely, and would still have the refrain, "from the candidate."

"Where the successful prosecution of a calling requires a certain amount of technical knowledge and professional skill, and the lack of them in the practitioner will result in material damage to the one who employs him, it is a legitimate exercise of police power to prohibit any one from engaging in the calling who has not previously been examined by the lawfully constituted authority and received a certificate in testimony of his qualification to practise the profession.

"It is the common custom in all the towns and cities of the United States to require the payment of a certain sum of money as a license fee, for the privilege of prosecuting one's profession or calling. The license is required indiscriminately of all kinds of occupations, whatever may be their character, whether harmful or innocent, whether the license is required for the protection of the public or not. . . . It is either a license, strictly so called, imposed in the exercise of the ordinary police power of the State, or it is a tax laid in the exercise of the power of taxation. In many cases it becomes exceedingly important to determine under which power the particular license is imposed. For example, if the license is a tax, the bill must originate in the House of Representatives, according to the almost universal requirements of constitutional law. But if it is a police regulation, the bill providing for it is constitutional in whichever house it was introduced.

"It is one of the 'ways and means' of defraying current expenses."[1]

A license tax has been held to be reasonable when imposed upon venders of milk, hucksters, pedlers, venders of cigarettes, upon attorneys, physicians, bankers, hacks and drays, and other vehicles. So, likewise, may such tax be exacted of keepers of places of amusement, of dealers in second-hand articles, pawnshops, insurance brokers, auctioneers. In short, the State has the power to impose a license fee as a tax or a police license upon every kind of business.

---

[1] Tiedeman's Police Regulation of Skilled Trades and Professions.

"In the regulation of all such occupations, it is constitutional to *require those who apply for a license to pay a reasonable sum* to defray the expenses of issuing the license, and what is a reasonable sum must be determined by the facts in the case.

"When a municipal corporation is given the power to license useful trades or occupations, it cannot use the license as a tax to raise revenue, nor is it authorized to entirely prohibit the exercise of the trade or occupation by any excessive license fee."[1]

Mr. Justice Manning says, "A proper license tax is not a tax at all within the meaning of the constitution or even within the ordinary signification of the word tax. . . . The imposition of a license tax is in the nature of the sale of a benefit, or privilege, to the party who would not otherwise be entitled to the same. The imposition of an ordinary tax is in the nature of the requisition of a contribution from that which the party taxed already rightfully possesses."

I have examined all State laws concerning dentistry, numerous other laws, and legal authorities, and so far I have not succeeded in finding a single case where the costs did not fall upon the applicant; therefore, in answer to the query, "How shall the revenue for conducting the work of State boards be obtained?" I reply, "From the candidate."

[1] American and English Encyclopædia of Law.

